# Treating acute lung injury through scavenging of cell-free DNA by cationic nanoparticles

**DOI:** 10.1016/j.mtbio.2024.101360

**Published:** 2024-11-25

**Authors:** Ziyan Huang, Cong Wei, Hanbin Xie, Xue Xiao, Tienan Wang, Yihan Zhang, Yongming Chen, Ziqing Hei, Tianyu Zhao, Weifeng Yao

**Affiliations:** a Department of Anesthesiology, The Third Affiliated Hospital, Sun Yat-sen University, Guangzhou510630, PR China; b School of Materials Science and Engineering, Sun Yat-sen University, Guangzhou 510275, PR China; c Key Laboratory for Polymeric Composite and Functional Materials of Ministry of Education, Sun Yat-sen University, Guangzhou 510275, PR China

**Keywords:** Cationic nanoparticles, acute lung injury, NETs, cfDNA, cGAS–STING

## Abstract

Acute lung injury (ALI) and acute respiratory distress syndrome are life-threatening conditions induced by inflammatory responses, in which cell-free DNA (cfDNA) plays a pivotal role. This study investigated the therapeutic potential of biodegradable cationic nanoparticles (cNPs) in alleviating ALI. Using a mouse model of lipopolysaccharide-induced ALI, we examined the impact of intravenously administered cNPs. Our findings indicate that cNPs possess robust DNA binding capability, enhanced accumulation in inflamed lungs, and a favorable safety profile *in vivo*. Furthermore, cNPs attenuate the inflammatory response in LPS-induced ALI mice by scavenging cfDNA, mainly derived from neutrophil extracellular traps, and activating the macrophage-mediated cGAS-STING pathway. The findings suggest a potential treatment for ALI by targeting cfDNA with cNPs. This approach has demonstrated efficacy in mitigating lung injury and merits further exploration.

## Graphical abstract

**Figure:**
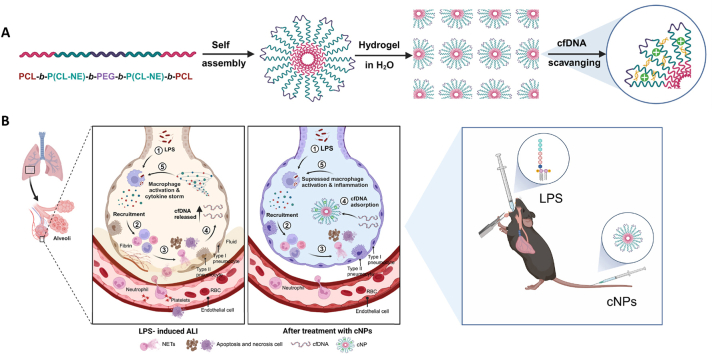

## 1 Introduction

Acute lung injury (ALI) and its severer counterpart, acute respiratory distress syndrome (ARDS), are highly prevalent and life-threatening respiratory conditions, holding mortality rates of approximately 35–45 % [1]. ARDS manifests as severe hypoxemia and pulmonary edema stemming from increased alveolocapillary permeability, essentially representing an inflammatory storm [2]. The current inadequacy of effective pharmacotherapy calls for novel therapeutic strategies for ALI/ARDS.

Cell-free DNA (cfDNA) is a short-lived, low-molecular-weight, double-stranded DNA found in limited quantities in the bloodstream [3]. During pathological processes, DNA fragments are released into circulation by apoptotic cells, dead cells, and neutrophil extracellular traps (NETs), ultimately forming cfDNA [4]. Indeed, cfDNA has been exploited as a noninvasive biomarker in liquid biopsies for various applications, including prenatal testing [5], cancer diagnosis [6], transplant rejection monitoring [7], and the tracking of microbial infections [8].

Recent data have revealed a close correlation between elevated cfDNA levels and acute-phase reactants, neutrophil counts, and illness severity [9]. Beyond serving as a biomarker for tissue injury, cfDNA acts as a damage-associated molecular pattern (DAMP), which can interact with pattern recognition receptors to activate innate immune signaling [10]. Several DNA sensors capable of recognizing cfDNA have been identified, including Toll-like receptor 9 (TLR9) [11] within endosomes and cytoplasmic sensors like cyclic GMP-AMP synthase (cGAS) [12] and absent in melanoma 2 (AIM2) [13]. The cGAS-stimulator of interferon genes (STING) pathway, a canonical cytosolic DNA sensor discovered in 2012 [14], plays a pivotal role in many inflammatory diseases as well as cellular senescence including autophagy and necroptosis [15].

DNA entering the cytoplasm activates cGAS and produces the second messenger cyclic GMP-AMP (cGAMP) to bind with the endoplasmic reticulum (ER)-localized adaptor protein STING. Activated STING translocates from the ER to the Golgi apparatus, where it recruits TBK1 to phosphorylate interferon regulatory factor 3 (IRF3), which then translocates to the nucleus to activate gene transcription of type-I interferons and other pro-inflammatory cytokines.

**Scheme 1:**
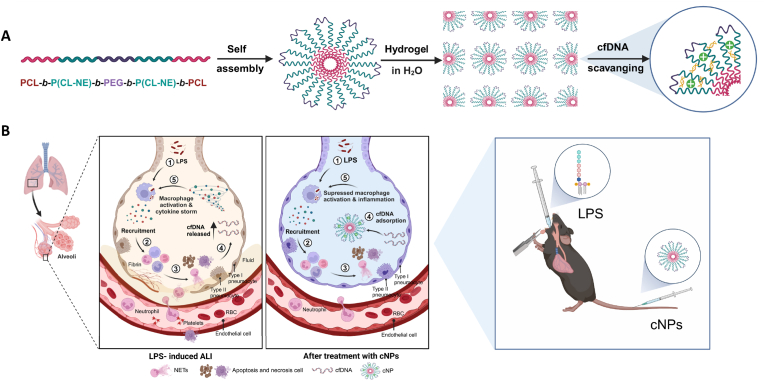
A schematic illustration detailing the synthesis of pentablock cNPs and their application in ALI therapy is provided.

Currently, cationic nanomaterials (cNPs) have shown great potential in the treatment of diseases. In 2011, Sullenger et al. proposed using cationic polymers to remove proinflammatory nucleic acids for therapeutic purposes [16]. We and others have previously reported that cNPs can scavenge the negatively charged cfDNA, in many animal models of inflammatory diseases including rheumatoid arthritis [17], systemic lupus erythematosus [18], acute liver failure [19], cancer metastasis [20], influenza infection [21], sepsis [22], and inflammatory bowel disease [23]. However, no prior studies have explored using cfDNA-scavenging cationic nanomaterials for mitigating damage in LPS-induced ALI. We hypothesized that the progression of lung injury would be accompanied by the release of a large number of NETs, which would cause an elevation of cfDNA levels. Therefore, we sought to test the hypothesis that targeting cfDNA represents a viable therapeutic strategy for alleviating ALI (see Scheme 1).

This study aims to investigate the interplay between cfDNA levels and the severity of LPS-induced ALI in mice, characterize the DNA-binding affinity and toxicity of cNPs, conduct *in vivo* validation experiments, and elucidate the immune regulatory mechanisms involved in cfDNA scavenging. Our results collectively support the hypothesis that nanoparticulate cfDNA scavengers offer promise in treating LPS-induced ALI.

## 2 Experiment section

### 2.1 Synthesizing the pentablock copolymers

We constructed a cationic block polymer by carrying out sequential ring-opening polymerization (ROP) reactions with α-bromo-ε-caprolactone (CL-Br) and then with ε-caprolactone CL. CL-Br monomers were synthesized via N-bromosuccinimide substitution with cyclohexanone and a subsequent Baeyer–Villiger oxidation reaction. Polyethylene glycol (PEG; number-average molecular weight = 4 kDa) was selected as the initiator for the ROP reaction with CL-Br. The product, P(CL-Br)_10_-*b*-PEG-*b*-P(CL-Br)_10_, was used as a microinitiator in an ROP reaction with CL, using diphenyl phosphate as a catalyst. Post-polymerization modification of PCL_10_-*b*-P(CL-Br)_10_-*b*-PEG-*b*-P(CL-Br)_10_-*b*-PCL_10_ with *N, N-*Diethyl-*N′*-methylethylenediamine (NE) was successfully conducted employing “click” chemistry, as shown by ^1^H NMR spectroscopy (AVANCE III 400 MHz, Bruker).

### 2.2 Agarose gel electrophoresis assay

Different concentrations of cNPs were prepared and mixed with 0.4 μL of CpG1826 solution (1 mg/mL) to obtain complex solutions with different N/P ratios, respectively, and incubated for 30 min at room temperature. The resulting solution was then added to a 1 % agarose gel containing the nucleic acid dye and run in TAE buffer at 80 V and 30 mA for 30 min. Final photographs were taken with a gel imaging system (Gel Doc XR+, Bio-Rad).

### 2.3 Fluorescence quenching

EtBr solution (3 μL, 1 mg/mL in PBS) was added to 3 mL Britton–Robinson buffer and mixed well. ctDNA (12 μL, 1 mg/mL in PBS) was then added. Different volumes of cNPs (1 mg/mL in deionized water) were added in succession. The emission spectrum of the solution was obtained from 500 to 800 with excitation at 280 nm.

### 2.4 Protein adhesion

Protein adhesion to cNPs was detected using the BCA Protein Assay Kit. Briefly, 50 μL of cNPs (1 mg/mL in PBS) was mixed with BSA solution (2 mg/mL). We also prepared BSA standards (100 μL, 0.03–2 mg/mL) via serial dilution. The samples and standards were incubated at 37 °C for 1 h with shaking, followed by centrifugation for 5 min at 8000 rpm. Then, each supernatant (20 μL) was transferred to a separate well of a 96-well plate, fresh mixed BCA working reagent (200 μL) was added to each well, and the wells were incubated at 37 °C for 30 min. Absorbance at 562 nm was measured with a multi-function enzyme labeler (Synergy 2, BioTeck, USA). Protein adhesion was calculated using the following relationship: *(C*_*C*_*-C*_*T*_*)V/m*, where *C*_*C*_ is the BSA concentration of the control sample (1 mg/mL), and *C*_*T*_ is the concentration of BSA after mixing with cNPs.

### 2.5 Mice

Male C57BL/6J mice (8 weeks old, 22–26 g) were purchased from Guangdong Sja Biotechnology Co., Ltd. (Guangdong, China). All mice were housed in a specified pathogen-free environment (22–26 °C, 12 h light/dark cycle) and had free access to water and food. The animal care and experiments were approved by the Ethics Committee of Guangdong Zhiyuan Biomedical Technology Co. (IAEC-2022081001).

### 2.6 Generating the mouse model of LPS-induced ALI and treatment

After anesthetizing with sodium pentobarbital (1 %, 50 mg/kg, i.p. injection), the upper teeth of mice were suspended on a 45° inclined plate. The tongue was picked up with forceps, and LPS (dissolved in PBS, 5 mg/kg, 25 μL) was injected into the tracheal with a quantitative nebulizer for lung fluids (Catalog No. YAN30012, Yuyanbio) after exposing the vocal folds under a mouse laryngoscope (Catalog No. SR310-RM, Yuyanbio). All mice were then placed vertically to ensure that LPS was evenly distributed in both lungs. After endotracheal LPS administration, the mice were immediately injected via the tail vein with deionized water or cNPs (dissolved in deionized water, 10 mg/kg, 100 μL) using a visual fixator.

### 2.7 Collecting BALF samples

The mice were immobilized supine on an operating table, and the main trachea was separated. Using ophthalmic scissors, a small incision was made in the trachea. Next, the trachea was inserted by a 22 G cannula needle and secured with 4-0 silk thread. The trachea was slowly flushed thrice with pre-cooled PBS (800 μL). The supernatant was collected and frozen after centrifugation (3000 rpm, 10 min, 4 °C).

### 2.8 cfDNA extraction and quantification

Serum, BALF, and culture supernatant samples were collected and centrifuged (3000 rpm, 10 min, 4 °C). The DNA purification kit was used to extract cfDNA according to the manufacturer's instructions. The cfDNA concentrations were determined using NanoDrop 2000 Spectrophotometers (Thermo Scientific).

### 2.9 NET staining

Frozen sections of lung tissue were returned to room temperature and fixed in 4 % paraformaldehyde for 30 min. The sections were blocked with 5 % goat serum for 30 min and then incubated with anti-histone H3 primary antibody (1:100) at 4 °C overnight and Alexa Fluor® 488 secondary antibody in the dark for 1 h. They were also labeled with anti-MPO antibodies (1:100) and Alexa Fluor® 594. At last, they were stained with DAPI and imaged under an inverted fluorescence microscope.

### 2.10 Assessing the biodistribution of cNPs

IR808-labeled cNPs (5 mg/kg, 100 μL) were administered intravenously to LPS model mice. A scalp needle and a systemic perfusion fluid with 0.9 % saline were inserted into the left ventricle of the mice to exclude interference with blood. Brain, heart, liver, spleen, lung, and kidney samples were obtained for in vitro fluorescence imaging at different time points during 72 h. The average fluorescence intensity was calculated using Living Image software (IVIS Spectrum, PerkinElmer Inc).

### 2.11 Cell culture and treatment

RAW264.7 macrophages were cultured in DMEM with 10 % FBS and 1 % penicillin-streptomycin (37 °C, 5 % CO2). cfDNA was extracted from mouse BALF and quantified. Equivalent (200 ng) cfDNA and poly(dA:dT) were individually transfected into cells with Lipofectamine 3000 following the manufacturer's protocol. After 4 h, the medium was removed and washed with PBS thrice and replaced with fresh medium containing cNPs (25 or 50 μg/mL). After 24 h, cell supernatants in 96-well plates were collected and detected by mouse ELISA TNF-α and IL-6 ELISA kits. The cells seeded in 6-well plates were collected to evaluate p-STING, STING, p-TBK1, and TBK1 levels via western blotting.

### 2.12 MTT assay

Cytotoxicity of cNPs was assessed by MTT assay using RAW264.7 macrophages. Cells were seeded overnight in 96-well plates. Next, different doses of cNPs were administered and incubated for 24 h. MTT (10 μL, 5 mg/mL) was then added to each well for 4 h. DMSO (100 μL) was replaced with supernatant, and the plates were shaken for 10 min in the dark. The absorbance of each well was recorded at 570 nm using an enzyme marker.

### 2.13 Western blotting

Mouse lung tissues (20 mg) and RAW264.7 cells seeded in 6-well plates were washed with PBS and added with 100 μL lysis buffer (RIPA: PMSF: phosphatase inhibitor = 100:1:1). They were then thoroughly ground by grinder and ultrasonic breaker (Sonics VCX105). The supernatant was obtained after centrifugation at 12,500 rpm for 15 min at 4 °C. Total protein concentration was measured by BCA protein assay. After electrophoresis with 10 % PAGE (40 μg/well), the proteins were transferred to PVDF membranes. The membranes were then blocked with 5 % skimmed milk powder for 1 h and incubated with primary antibodies against cGAS (1:1000), STING (1:1000), TBK1 (1:1000), p-STING (1:500) and p-TBK1 (1:500) at 4 °C overnight. Anti-GAPDH (1:10000) primary antibody and goat anti-rabbit IgG H&L (HRP) secondary antibody were separately incubated for 1 h. Bands were exposed using a chemiluminescence imager (ImageQuant LAS 500), and gray values were quantified using ImageJ software.

### 2.14 Statistical analysis

SPSS, GraphPad Prism 9.0, and MatlabR2022b software performed all statistical analyses. Data were expressed as mean ± SE. Comparisons between groups were analyzed by unpaired two-tailed Student's t-test or one-way ANOVA when appropriate. Correlation analysis was performed by linear regression. *p* ≤ 0.05 was considered statistically significant.

## 3 Results

### 3.1 cfDNA level correlated positively with disease severity and NET formation in LPS-induced ALI

**Fig. 1:**
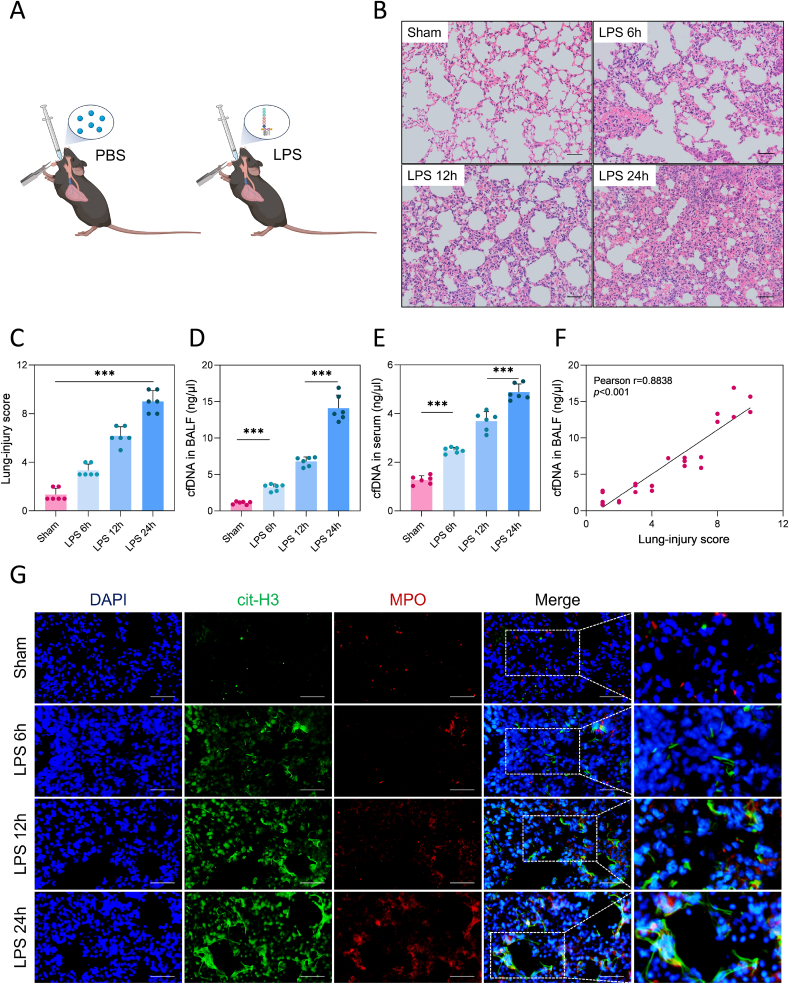
**cfDNA correlated positively with disease severity and NET formation in LPS-induced ALI. (A)** Schematic diagram of intratracheal injection of PBS or LPS in mice. **(B, C)** Representative H&E-stained slices demonstrating the pathological injury scores of the mouse lung tissues. Scale bars: 100 μm, n = 6/group. **(D, E)** The BALF and serum cfDNA levels in mice at 6, 12, and 24 h after LPS treatment, n = 6/group. **(F)** A positive correlation was found between BALF cfDNA levels and lung-injury scores. **(G)** Representative IF-staining images of NETs from frozen mouse lung tissue sections. Immunostaining for cit-H3 (green), MPO (red), and nuclei (blue). Scale bars: 100 μm. The data shown are mean ± SE (*∗∗∗p* < 0.001).

Prior studies have demonstrated that circulating cfDNA can effectively predict outcomes in inflammatory lung disease and sepsis [24,25]. To delve deeper into the role of cfDNA in ALI, we established a mouse model of ALI via intratracheal injection of LPS (Fig. 1A) [26]. The mice were euthanized at 6, 12, or 24 h following LPS exposure. We assessed histological alterations in lung tissues via hematoxylin and eosin (H&E) staining. LPS instillation significantly exacerbated inflammatory cell infiltration in the alveoli (Fig. 1B), and the lung injury score increased over time after LPS administration (*p* < 0.001, Fig. 1C), with the peak being observed at 24 h after treatment.

Simultaneously, these results were in line with other assessments, such as Evans Blue dye [27] (Fig. S1A) and terminal deoxynucleotidyl transferase-mediated nick-end labeling (TUNEL) staining (Fig. S1B) in lung tissues, indicating the progressive nature of lung injury over time. We found that the lung tissues of mice in the model group deepened in blue staining with time, and the concentration of Evans blue gradually increased. This suggests that stimulation of LPS impaired the alveolar-capillary barrier, leading to increased permeability. Likewise, the green fluorescence of the LPS model group was gradually enhanced, indicating that apoptosis of lung tissues gradually increased.

What's interesting, we explored the trends of cfDNA levels within serum and bronchoalveolar lavage fluid (BALF) samples. As expected, the mice with ALI exhibited higher cfDNA levels than their healthy counterparts. At 6 h post-LPS treatment, cfDNA levels began to rise and kept increasing for the post-24 h (*p* < 0.001, Fig. 1D and E). The cfDNA concentration in the BALF samples was three-fold higher than in the serum samples. Moreover, the BALF cfDNA levels correlated positively with the lung injury scores, according to Pearson correlation analysis (r = 0.8838; Fig. 1F).

To further gain insights into the origin of cfDNA in BALF, we analyzed frozen mouse lung-tissue sections via immunofluorescence (IF). The results revealed that LPS treatment led to an increase in the amount of citrullinated histone H3 (cit-H3) and myeloperoxidase (MPO) double-positive neutrophils in the ALI group over time, indicating a potential escalation in the neutrophil extracellular trap (NET) formation [28] (Fig. 1G). Consequently, we selected the 24 h time point for intervention in subsequent mouse experiments.

### 3.2 Synthesis and characterization of the pentablock copolymer and its self-assembled nanoparticles

**Fig. 2:**
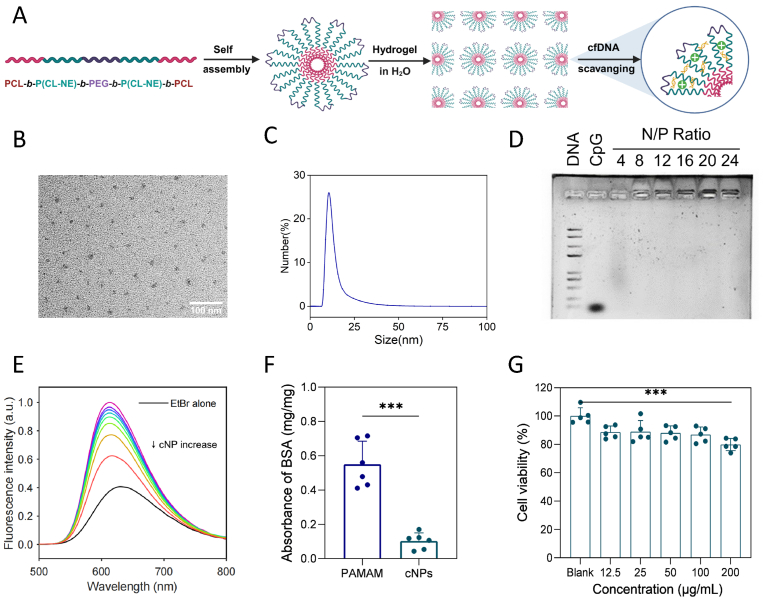
**Synthesis and characterization of pentablock cNPs. (A)** Schematic diagram of the synthesis and action of cNPs. **(B)** TEM image of cNPs after incubation in deionized water. Scale bar: 100 nm. **(C)** Particle-size spectrum, as determined by DLS. **(D)** Agarose gel electrophoresis-retardation assay for cNPs-CpG 1826 complexes. **(E)** Fluorescence spectra of EtBr bound to ctDNA in the presence of different concentrations of cNPs in PBS. **(F)** Adsorption of BSA to PAMAM or cNPs after co-incubation. **(G)** Cell viability in RAW264.7 macrophages after incubation with different doses of cNPs. The data were represented by mean ± SE (*∗∗∗p* < 0.001).

In this work, we synthesized cationic nanoparticles (cNPs), based on pentablock polyethylene glycol (PEG)/polycaprolactone (PCL)-copolymers modified by cationic moieties. Hydrophilic PEG blocks formed the outer shell and hydrophobic PCL blocks aggregated with each other to form the core, which self-assembly formed a "fancy" micellar structure (Fig. 2A). The synthesis of the amphiphilic copolymers involved four key steps: (I) the synthesis of α-bromo-ε-caprolactone (CL-Br) monomer, (II) the synthesis of the triblock P(CL-Br)_10_-*b*-PEG-*b*-P(CL-Br)_10_, (III) the synthesis of the pentablock PCL_10_-*b*-P(CL-Br)_10_-*b*-PEG-*b*-P(CL-Br)_10_-*b*-PCL_10_, and (IV) the synthesis of final product PCL_10_-*b*-P(CL-NE)_10_-*b*-PEG-*b*-P(CL-NE)_10_-*b*-PCL_10_. *N, N*-Diethyl-*N′*-methylethylenediamine (NE) was selected as the cationic species employing “click” chemistry [29], as illustrated in Fig. S2A.

The ^1^H NMR spectra showed the successful synthesis of the intermediate product (Figs. S2B, C, D). The hydrogen atom adjacent to the bromine atom (f peak) shifted from the 4.5 ppm to the 3.2 ppm position, indicating that the bromine atom was completely replaced (Fig. S2E). Fourier transform infrared (FTIR) was also depicted (Fig. S3A), showing the successful addition of monomers. The gel-permeation chromatography (GPC) traces (Fig. S3B) showed that molecular weight was in line with the designed degree of polymerization, and the unimodal peak indicates a narrow size distribution with a polydispersity index *Đ* < 1.3. The excitation and emission spectra and GPC curves of IR808 fluorescently labeled cNPs are shown in Figs. S3C and D.

The morphology of cNPs was observed under transmission electron microscopy (TEM), showing the roughly uniform size of cNPs and excellent dispersion. The size of cNPs is estimated to be below 30 nm. (Fig. 2B). The hydrodynamic diameter of cNPs was further measured by dynamic light scattering (DLS). The size, polydispersity index, and ζ-potential of cNPs were 19.13 ± 5.85 nm, 0.31 ± 0.01, and 26.81 ± 2.27 mV, respectively (Fig. 2C).

Agarose gel electrophoresis was used to investigate the binding capability of cNPs as a CpG scavenger. Gel-retardation assays showed that the cNPs impeded the migration of free CpG molecules toward the cathode, indicating the complete complexation of CpG with cNPs at an N: P ratio＞4 (Fig. 2D). In ethidium bromide (EtBr)-competition assays (Fig. 2E), the black line indicates that only EtBr is present in the solution, and the spectrum rises to the highest level after the addition of calf thymus DNA (ctDNA). The colored lines from top to bottom indicate that as the concentration of cNPs increases, it competes to bind DNA, causing EtBr decomplexation to become free and significantly decreasing the fluorescence intensity.

Protein absorption of cNPs can disturb their capacity to bind DNA; thus, their capacity to resist protein attachment is essential for the system. Bovine serum albumin (BSA) was used as a model protein to simulate the interaction of cNPs with proteins *in vivo*. As shown in Fig. 2F, after incubating cNPs with BSA for 1 h in PBS, ∼0.1 mg/mg of BSA was absorbed into the cNPs. The BSA-binding capacity was only about 1/5 that of polyamidoamine (PAMAM) dendrimer, which exhibited strong resistance to protein binding (*p* < 0.001). Moreover, the cell viability of cNPs was evaluated in RAW264.7 macrophages via 3-(4,5-dimethyl-2-thiazolyl)-2,5-diphenyl-2-*H*-tetrazolium bromide (MTT) assays (Fig. 2G). Cell viability remained at 80 % when the concentration of cNPs reached 200 μg/mL, primarily attributed to the enhanced PEG-shielding effects (*p* < 0.001).

### 3.3 cNP injection decreased cfDNA levels and protected mice against LPS-induced ALI

**Fig. 3:**
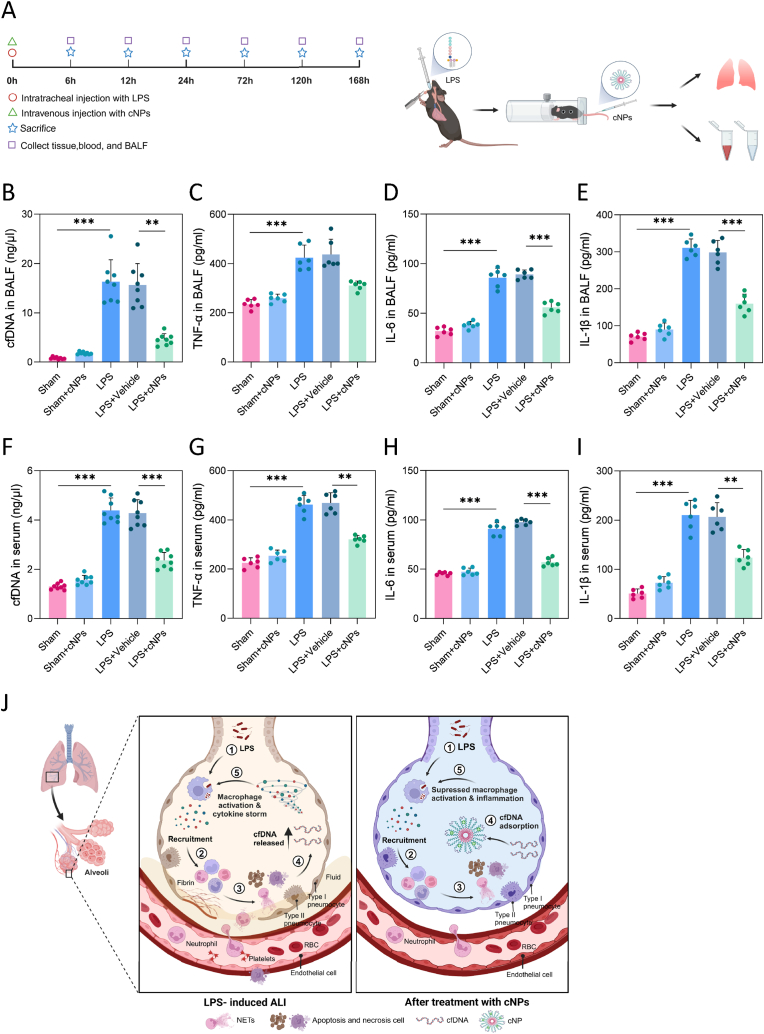
**cNP injection decreased the level of cfDNA and inflammatory factors in LPS-induced ALI. (A)** Timeline followed when administering LPS and cNPs and collecting tissues, blood, and BALF from mice. **(B, C, D, E)** The cfDNA, TNF-α, IL-6, and IL-1β levels of BALF in the indicated five groups at 24 h post-treatment. **(F, G, H, I)** The cfDNA, TNF-α, IL-6, and IL-1β levels of serum at 24 h post-treatment. **(J)** Diagram of the mechanisms associated with LPS-induced ALI, with or without cNP intervention. The data were represented by mean ± SE (*∗∗p* < 0.01, *∗∗∗p* < 0.001), n = 6–8/group.

To assess the efficacy and safety of cNPs in animals, we first induced ALI in mice through intratracheal LPS injection, followed by tail vein administration of cNPs. The mice were euthanized at intervals of 1, 3, 5, or 7 days under anesthesia, and tissue specimens were collected (Fig. 3A).

In the study we found the expression of cfDNA and inflammatory factors in BALF and serum after cNPs injection alone in healthy mice were slightly higher than the control group, indicating less inflammatory stimuli. The results unequivocally demonstrated that cNP administration significantly reduced cfDNA levels in both BALF and serum, underscoring the robust DNA-binding capacity of cNPs (*p* < 0.01, Fig. 3B–F). Additionally, cNP injection declined LPS-induced release of inflammatory factors, as evidenced by decreased levels of tumor necrosis factor (TNF)-α (Fig. 3C–G), interleukin (IL)-6 (Fig. 3D–H), and interleukin (IL)-1β (Fig. 3E–I) in both BALF and serum samples. The diagram showed the mechanism of cfDNA-mediated pro-inflammatory responses in LPS-induced ALI and cNPs attenuate the inflammatory response by scavenging cfDNA (Fig. 3J).

**Fig. 4:**
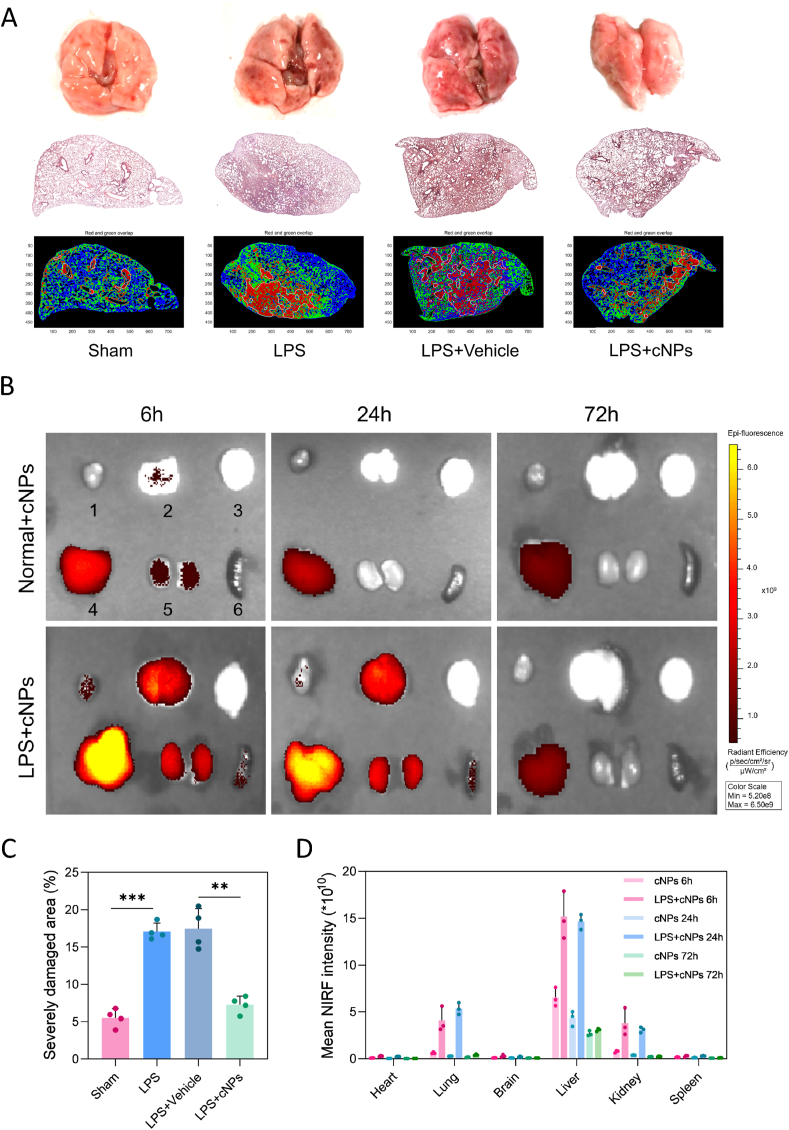
**cNPs reduced lung damage and led to an EPR effect. (A)** Lung taking pictures and lung damage tracing based on H&E staining, n = 4/group. **(B)** Ex vivo near-infrared fluorescence (NIRF) images of different organs (1: heart, 2: lung, 3: brain, 4: liver, 5: kidney, and 6: spleen) from normal mice and mice with ALI at 6, 24, and 72 h after intravenous injection of IR808-labeled cNPs, n = 3/group. **(C)** Statistics of the red areas according to lung-damage tracing technique (blue: normal lungs; green: damaged lungs; red: severely damaged lungs). **(D)** Statistics of the mean fluorescence intensity of the organs. The data were represented by mean ± SE (*∗∗p* < 0.01, *∗∗∗p* < 0.001).

What's more, cNP injection mitigated LPS-induced overall lung-tissue lesions. With the naked eye, we can observe that the surface of the lung tissue in the material group was lighter in color and had fewer hemorrhagic spots. The whole H&E stained sections were analyzed for lung tissue damage tracing [30] and found that cNPs protected mice against ALI, as evidenced by reduced red zones with severely damaged lung tissues (*p* < 0.01, Fig. 4A). H&E staining also revealed that inflammatory cell infiltration in the lungs triggered by LPS, was attenuated by cNP administration (Figs. S4A and B). Similarly, TUNEL-staining results demonstrated a decrease in apoptosis in lung tissues in the presence of cNPs (Figs. S4C and D).

### 3.4 cNPs have a lung-targeting effect

To probe the biodistribution of cNPs *in vivo*, we synthesized IR808 fluorescently labeled cNPs, administered intravenously. Fluorescence imaging showed a peak signal in various organs within 24 h, with a subsequent rapid decline, indicating metabolism within 72 h (Fig. 4B–D). The ALI mice injected with cNPs exhibited stronger fluorescence signals in the lungs, liver, and kidneys compared to normal mice. Minimal fluorescence signals were observed in the heart, brain, and spleen. Importantly, the lung tissue of ALI mice showed a five-fold higher fluorescence signal than normal mice at 24 h, suggesting that cNPs have a lung-targeting effect.

This is because the increased vascular permeability of the lungs in the pathological ALI environment may contribute to nanoparticle-mediated passive targeting delivery [31], thereby leading to an enhanced permeability and retention (EPR) effect [32] in the damaged lung tissues. The EPR effect, which manifests itself as the passive delivery of biomaterial-drug agents to the site of inflammation through a leaky vascular system around the inflamed tissue, was first established in the development of anticancer nanomedicines.

To assess the toxicity of cNPs in systemic organs, histopathological and biochemical analyses were performed on mice treated exclusively with five times the dose of cNPs over 7 days. We anesthetized the mice on days 1, 3, 5, and 7 and collected their organs and blood. In the mice, H&E-stained sections revealed minimal inflammatory cell infiltration in the heart, spleen, liver, lungs, and kidney, showing mild organ injuries compared to healthy mice (Fig. S5A). TUNEL results also showed minimal apoptosis in the systemic organs (Fig. S5B). Consistent with these histological findings, serum alanine aminotransferase (ALT), aspartate aminotransferase (AST), urea nitrogen (Urea), and creatinine (Cr) indicate that the cNPs had little impact on liver function and kidney function (Fig. S5C). The above results indicated that cNPs have a good safety profile *in vivo*.

### 3.5 cNPs inhibited NET formation and cGAS-STING pathway expression in the lung

**Fig. 5:**
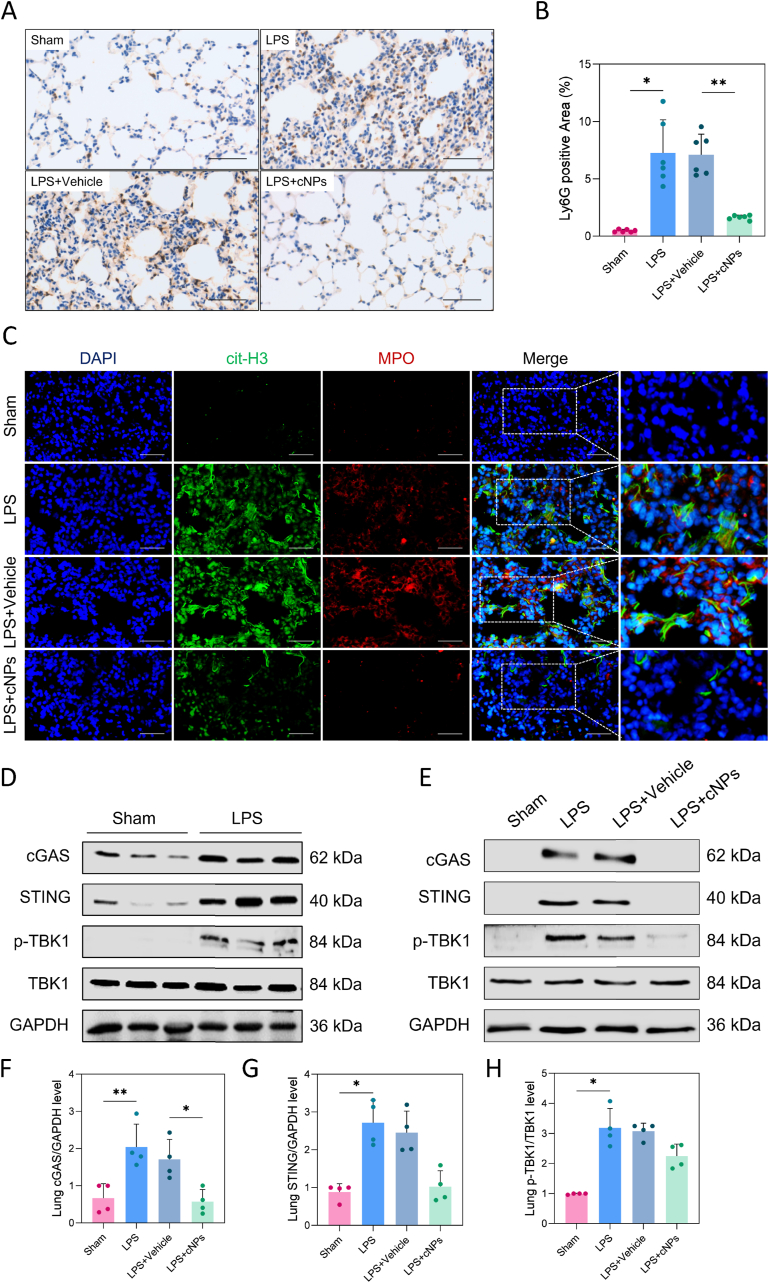
**cNPs inhibited NET formation and cGAS-STING pathway expression in the lung. (A, B)** IHC staining of lung tissues for Ly6G and statistics of positive areas (yellowish-brown color). Scale bars: 100 μm, n = 6/group. **(C)** IF staining of NETs from frozen lung tissue slices. Scale bars: 100 μm, n = 3/group. **(D)** The protein expression levels of cGAS, STING, p-TBK1, and TBK1 in lung tissues of two groups by western blotting, n = 3/group. **(E, F, G, H)** Relative protein expression levels of the cGAS-STING pathway in the four lung tissue groups and statistics of the grayscale ratio (fold change to Sham group), n = 4/group. The data were represented by mean ± SE (*∗p* < 0.05, *∗∗p* < 0.01).

To unravel the mechanism behind the efficacy of cNPs in mitigating LPS-induced ALI, we focused on the immune regulation of neutrophils to decipher the patterns of cfDNA trafficking. Immunohistochemical (IHC) staining revealed increased Ly6G expression following LPS exposure (Fig. 5A and B), indicating the recruitment of numerous activated neutrophils to the alveoli. Additionally, IF staining of frozen lung tissue sections demonstrated that LPS treatment led to an elevated expression of both cit-H3 and MPO in the neutrophils, suggesting increased NET formation (*p* < 0.05, Fig. 5C, Figs. S6A and B). The phenomenon aligns with the concept that neutrophils employ NETs to combat invading pathogens by ensnaring and neutralizing them [33]. However, cNP administration effectively reversed the exacerbated lung damage, as indicated by reduced Ly6G expression and diminished NET formation. These findings underscore the effect of cNPs in attenuating LPS-induced ALI by restraining neutrophil infiltration and limiting NET formation.

Activation of the cGAS-STING-signaling axis, known for its role in promoting inflammation, has been implicated in various acute and chronic lung disease [34,35], which most likely involves cellular stress responses to infection. To determine whether the cGAS-STING pathway was triggered by LPS treatment, we first evaluated the expression levels of key proteins within the cGAS-STING-signaling axis in lung tissues. Our observations revealed a consistent elevation in the levels of cGAS, STING, and phosphorylated TANK-binding kinase 1 (p-TBK1) following LPS administration (*p* < 0.05, Fig. 5D, Figure S6C, D, E). However, these changes were reversed by cNP treatment. As seen in the material group, the expression of these proteins was significantly attenuated and close to that of normal tissues (Fig. 5E, F, G, H)., indicating that the expression of the cGAS-STING pathway was markedly inhibited.

### 3.6 cNPs suppressed the activation of the STING pathway mediated by internalized cfDNA in RAW264.7 macrophages

STING proteins are mainly expressed in macrophages, dendritic cells, epithelial cells, and endothelial cells, associated with cell apoptosis, necrosis, and senescence. Macrophages, as pivotal initiators of inflammatory responses, serve as central regulators in immune regulation [36]. F4/80 represents one of the primary macrophage markers, and the expression of F4/80 significantly increased stimulated by LPS, an effect that was subsequently reversed by additional treatment with cNPs (Figs. S6F and G).

**Fig. 6:**
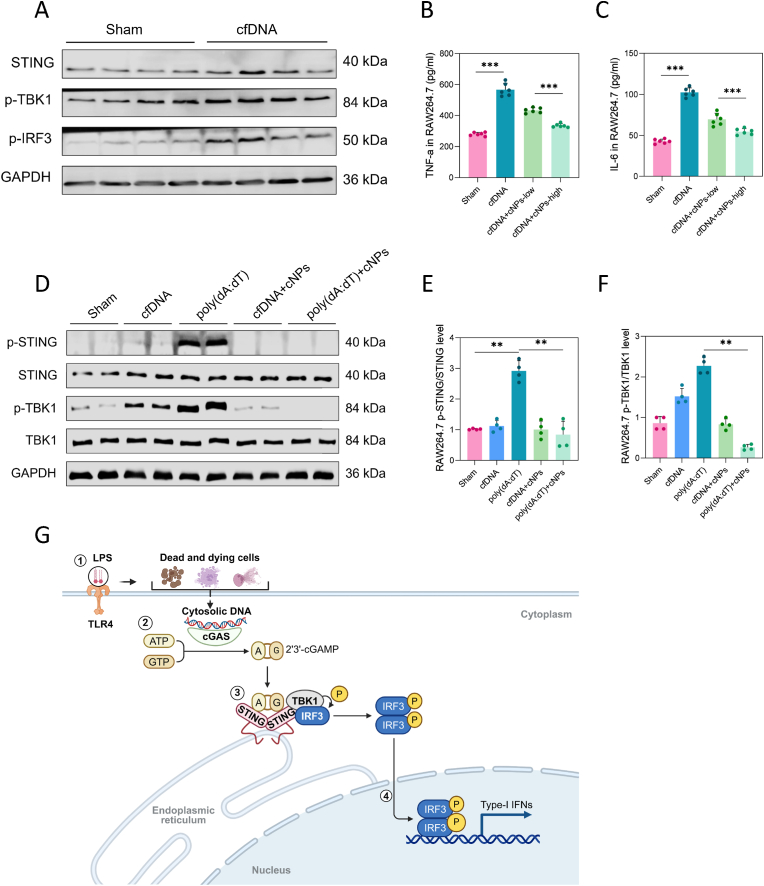
**cNPs suppressed the activation of the STING pathway mediated by internalized cfDNA in RAW264.7 macrophages. (A)** The protein expression levels of STING, p-TBK1, and p-IRF3 in RAW264.7 macrophages after transfecting with cfDNA, n = 4/group. **(B, C)** The TNF-α and IL-6 levels of cell supernatant were detected by ELISA, n = 6/group. **(D)** Relative protein expression levels of p-STING, STING, p-TBK1, and TBK1 of five groups in macrophages after stimulating with cfDNA or poly(dA:dT), with or without cNPs. **(E, F)** Statistics of the grayscale ratio of p-STING/STING and p-TBK1/TBK1 in macrophages (fold change to Sham group), n = 4/group. **(G)** Schematic representation of the main source of cytosolic cfDNA and the activation of the cGAS-STING pathway in the cytoplasm. The data were represented as mean ± SE (*∗∗p* < 0.01, *∗∗∗p* < 0.001).

To confirm the pro-inflammatory role of cfDNA following LPS stimulation, we extracted and purified cfDNA from BALF. Transfection of cfDNA into RAW264.7 macrophages by Lipo3000 and continuous stimulation of cells for 24 h resulted in cfDNA internalization. STING, p-TBK1, and p-IRF3 protein expression were upregulated after cfDNA stimulation, indicating that cfDNA was successfully transfected (*p* < 0.05, Fig. 6A, Figure S7A, B, C). We then assessed the expression of inflammatory factors through enzyme-linked immunosorbent assays (ELISA). The level of TNF-α and IL-6 in cell supernatants was elevated, yet was dose-dependently suppressed by cNP treatment (Fig. 6). We also observed that cNPs exhibited more potent inhibition of cfDNA-induced inflammation at a higher concentration (50 μg/mL) than at a low concentration (25 μg/mL) (*p* < 0.001).

To assess the ability of internalized cfDNA to activate the STING pathway, we employed poly(dA:dT), a synthetic dsDNA sequence, as a positive control [37], which is detected by cytosolic DNA sensors like cGAS and triggers the production of type-1 interferons. RAW264.7 macrophages were incubated with cfDNA or poly(dA:dT) to study the effects by western blotting. We found that transfected with either cfDNA or poly(dA:dT) led to increased expression of p-TBK1 and p-IRF3 (*p* < 0.05, Figs. S7D, E, F). Meanwhile, poly(dA:dT) group expressed greater proteins than the cfDNA one, as expected, given its ability to directly activate the STING pathway.

Interestingly, we found that internalized poly(dA:dT) induced a more than two-fold increase in p-STING and p-TBK1 production. However, incubation with cNPs (50 μg/mL) effectively blocked cfDNA-mediated activation of the STING pathway, regardless of whether stimulated with cfDNA or poly(dA:dT) (*p* < 0.01, Fig. 6D, E, F). This comprehensively confirmed that cNPs suppressed the activation of the STING pathway mediated by internalized cfDNA in RAW264.7 macrophages. Schematic illustration revealed the mechanism involved in the activation of intracellular cGAS-STING signaling pathway by cfDNA (Fig. 6G).

## 4 Discussion

ALI and ARDS, acute and serious respiratory diseases, are the main causes of death in critically ill patients; however, the underlying pathological mechanisms remain unclear. Although some researchers have successfully suppressed lung injury inflammation to a certain extent by using ROS scavenging [38,39] and pro-inflammatory factor inhibition [40,41], effective and practical treatments for ALI/ARDS are still lacking. Previous research showed that cfDNA acts as a biomarker of tissue damage and also a kind of DAMP that worsens systemic diseases characterized by exacerbated inflammation [42]. However, the link between cfDNA and ALI has not been reported. In this study, we demonstrated that the serum and BALF cfDNA levels increased significantly following LPS-induced ALI (especially in BALF), which was positively associated with the severity of lung injury.

Macrophages are the workhorses of the innate immune system and play a central role in immune defense and tissue repair. By interacting with TLR4, LPS activates macrophages to release pro-inflammatory cytokines and chemokines that recruit neutrophils from the bloodstream to the infection site. Neutrophils capture and kill bacteria through the formation of NETs. NETs are intricate extracellular mesh structures framed by depolymerized DNA strands and suffixed with cit-H3, NE, and MPO. Excessive apoptosis, necrosis, and NETosis explained the increased cfDNA levels in LPS-induced ALI, which in turn aggravated inflammatory response and thus constituted a positive feedback cycle. Our results underscored the significant role of cfDNA accumulation in the development of LPS-induced ALI.

To further evaluate the proinflammatory potential of cfDNA, we transfected RAW264.7 macrophages with BALF cfDNA and found that internalized cfDNA significantly activated the STING pathway. Poly(dA:dT) resulted in greater expression of phosphorylated proteins of the STING pathway may be due to its greater purification and the fact that it contains a high concentration of specific DNA sequences. However, the addition of high concentrations of cationic nanoparticles inhibited the cfDNA-mediated expression of the cGAS-STING pathway and proinflammatory-cytokines in macrophages, whether stimulated with cfDNA or poly(dA:dT). Consequently, STING activation is a crucial step in the progression of cfDNA-induced inflammation.

Scavenging cfDNA with cNPs has been demonstrated therapeutic effect in other inflammatory diseases, such as sepsis [22]. This strategy might be potentially viable for treating ALI. Therefore, we designed and synthesized biodegradable pentablock cNPs as cfDNA scavengers. We chose PEG(4000) instead of PEG(2000) as the shell to make its main chain longer, which not only reduces cytotoxicity but also improves the utilization of amine groups. When nanoparticles interact with proteins in the bioenvironment, they are surrounded by a layer of biomolecules called “protein corona”. The formation of the protein corona impairs the nano drug-targeting capability, and such nanoparticles can easily be engulfed by the reticuloendothelial system (RES) [43]. The five-block structure of nanoparticles with PEGylation of the shell and binding of nucleic acids in the middle segment proved to be very helpful in reducing nonspecific protein adhesion and prolonging the half-life of nanoparticles in circulation *in vivo* [44].

In ALI/ARDS, inflammatory mediators are “out of control”, causing apoptosis of the cells that make up the blood-air barrier, and in turn, increasing vascular permeability, which contributes to the nanoparticle-mediated lung-targeting effect. In general, medium-sized (20–200 nm) nanoparticles are the best for passively targeting inflamed tissues [31]. Therefore, intravenous injection circulating in the blood may be a more rational way to promote the co-localization of cNPs with damage-associated cfDNAs, due to an EPR effect in the lung. Besides, we found that cNPs tend to accumulate in the liver. Due to the abundant blood flow in the liver, which accounts for 10–15 % of the whole body, and the fact that Kupffer cells also remove circulating nanoparticles, this may reduce the efficiency of treatment and even affect liver function [45].

Although we obtained some exciting results in this study, some unclear issues remain that require future investigation. First, the difference in the specific sequences of BALF cfDNA and serum cfDNA might contribute to the mechanism of cfDNA clearance in ALI. Furthermore, the function of other DNA sensors besides cGAS, such as TLR9 and AIM2 in ALI, requires more research. Finally, the role of cfDNA as a proinflammation biomarker needs to be verified with clinical specimens from patients with ARDS.

## 5 Conclusion

Our research revealed a substantial increase in serum and BALF cfDNA levels in LPS-induced ALI, which correlated positively with disease severity. The accumulation of cfDNA induced by NET formation led to an over-activation of macrophages-mediated cGAS-STING pathway *in vivo* and in vitro. In this work, we harnessed degradable cNPs for treating LPS-induced ALI. Compared to traditional cNPs, these pentablock cNPs exhibited an enhanced cfDNA binding capacity, lower toxicity, and lung targeting effect. In summary, our study sheds light on the proinflammatory role of cfDNA in LPS-induced ALI and provides a potential therapeutic approach for treating ALI/ARDS using cationic polymer-mediated cfDNA scavenging.

## CRediT authorship contribution statement

**Ziyan Huang:** Writing – original draft, Methodology, Project administration. **Cong Wei:** Software, Methodology. **Hanbin Xie:** Methodology, Formal analysis. **Xue Xiao:** Methodology, Data curation. **Tienan Wang:** Investigation. **Yihan Zhang:** Writing – review & editing. **Yongming Chen:** Resources, Data curation. **Ziqing Hei:** Supervision, Funding acquisition. **Tianyu Zhao:** Writing – review & editing, Data curation, Conceptualization. **Weifeng Yao:** Writing – review & editing, Funding acquisition, Conceptualization.

## Consent for publication

All authors have read the manuscript and provided their consent for the submission.

## Declaration of competing interest

The authors declare that they have no known competing financial interests or personal relationships that could have appeared to influence the work reported in this paper.

## Data Availability

Data will be made available on request.
