# Supplementary material for: Treating acute lung injury through scavenging of cell-free DNA by cationic nanoparticles
Source: Mater Today Bio. 2024 Nov 25;29:101360. doi: 10.1016/j.mtbio.2024.101360 (PMC11648789; doi:10.1016/j.mtbio.2024.101360)
Supplement: Multimedia component 1 [file mmc1.docx]

**Supporting Information for**

Treating Acute Lung Injury through Scavenging of Cell-Free DNA by Cationic Nanoparticles

*Ziyan Huang^a,1^, Cong Wei^b,c,1^, Hanbin Xie^a,1^, Xue Xiao^a^, Tienan Wang^a^, Yihan Zhang^a^, Yongming Chen^b,c^, Ziqing Hei^a^, Tianyu Zhao^b,c,*^,Weifeng Yao^a,*^*

*^a^ Department of Anesthesiology*, *The Third Affiliated Hospital, Sun Yat-sen University, Guangzhou510630, PR China*

*^b^ School of Materials Science and Engineering, Sun Yat-sen University, Guangzhou 510275, PR China*

*^c^ Key Laboratory for Polymeric Composite and Functional Materials of Ministry of Education, Sun Yat-sen University, Guangzhou 510275, PR China*

*^1^These authors contributed equally to this work.*

**Corresponding Authors:*

*TZ (zhaoty25@mail.sysu.edu.cn); WY (yaowf3@mail.sysu.edu.cn)*

**Supplemental Methods**

*1. H&E Staining and Lung Injury Score*

Lung tissues fixed by 4% paraformaldehyde were used for paraffin embedding and sectioning. After the sections were dewaxed and rehydrated, they were stained by hematoxylin for 5 min. Next, they were immersed in hematoxylin differentiation solution and hematoxylin return blue solution. After alcohol dehydration, eosin was used to stain for 5 min. Finally, the sections were dewaxed, dehydrated, and sealed. The pathological changes of lung tissue were observed under an optional microscope (Vectra, PerkinElmer Inc). Lung edema, alveolar and septal inflammation, alveolar and interstitial hemorrhage, pulmonary atelectasis, and hyaline membrane formation were semi-quantitatively analyzed using the Smith score method, on a scale of 0-4, respectively. No injury was scored as 0, lesion extent <25% was scored as 1, 25%-50% was scored as 2, 50%-75% was scored as 3, and >75% was scored as 4. The total damage score was the sum of the above.

*2. Evans Blue-Leakage Analysis*

After intratracheal injection of LPS for 23 h, 200 μL Evans Blue solution (0.5% [w/v]) was injected through the mouse tail vein. Mice were euthanized 1 h later, and the thoracic cavity was opened. Lung tissues were collected, photographed, and weighed. Formamide (500 μL) was added to each tissue sample tube, followed by a water bath at 55℃ for 48 h. The supernatant was obtained by centrifugation, and the absorbance at 620 nm was measured by an enzyme marker. The amount of Evans blue exuded per mg of lung tissue was calculated.

*3. TUNEL Staining*

Paraffin sections of lung tissue were deparaffinized, rehydrated, and incubated with 100 μL proteinase K working solution for 20 min. After 20 min of membrane-breaking, the slices were incubated with 50 μL equilibration buffer. After 20 min, 56 μL labeling solution (recombinant TdT enzyme: FITC-12-dUTP labeling mix: equilibration buffer = 1:5:50) was added and incubated in the dark for 2 h. The nuclei were stained with 4,6-diamidino-2-phenylindole (DAPI), and sections were photographed under an inverted fluorescence microscope (Olympus, CKX41).

*4. IHC Staining*

After dewaxing and rehydrating, sodium citrate buffer was used to repair the antigen of paraffin sections, which were then blocked with 10% goat serum and incubated with primary antibodies against Ly6G (1:1000) or F4/80 (1:1000) overnight at 4 °C. Next, sections were immersed in 3% H_2_O_2_ and then added with anti-HRP for 15 min. Diaminobenzidine dye and hematoxylin were individually used for chromogenic reaction and nuclei staining. Finally, sequential dehydration, transparency, and sealing were performed. Pictures were photographed under an optional microscope and analyzed by ImageJ software.

*5. Preparing the cNPs*

cNPs were dissolved in 1 mL tetrahydrofuran to a concentration of 100 mg/mL. The resulting solution was added dropwise into sodium acetate buffer solution (10 mL, pH = 5.0) with vigorous stirring, stirred for over 0.5 h, transferred to a dialysis bag (Mn = 3500 Da), and dialysis against deionized water. The deionized water was renewed thrice for every 1 h of dialysis. The final cNP concentration was determined by lyophilizing. The size distribution and ζ-potential were characterized using DLS and ζ-nanosizer instruments (ZEN3600, Malvern, UK). TEM was used to take pictures of particles (JEOL1400 plus, Nippon Electronics Corporation).

*6. Fluorescently Labeling the Polymer*

Cationic polymer (500 mg) was dissolved in 0.5 mL dichloromethane (DCM), and IR808 (200 mg) was added. Next, 4-dimethylamino-pyridine (4 mg) was added, and 1-(3-Dimethylaminopropyl)-3-ethylcarbodiimide hydrochloride (10 mg) was eventually added to start the esterification. The reaction was allowed to stir in the dark for 1 h. The reaction product was precipitated in diethyl ether thrice and dissolved in DCM after each precipitation step.

Supplementary Figures

**Figure S1.** **The severity of lung injury progressively worsened over time in LPS-induced ALI. (A, B)** Representative pictures and determinations of Evans Blue (EB) concentrations in lung tissues at 1 h after tail vein injection with EB solution for LPS modeling. n = 6/group. **(C, D)** Representative TUNEL-staining images of mouse lung tissues and semi-quantitative analysis of TUNEL-positive areas. Scale bars: 100 μm, n = 6/group. The data shown represent the mean ± SE (***p* < 0.01, ****p* < 0.001).

**Figure S2. Synthesis and characterization of the cationic pentablock copolymer and its self-assembled nanoparticles. (A)** Synthesis routine for preparing cNPs. **(B)** ^1^H NMR spectrum of CL-Br. **(C)** ^1^H NMR spectrum of triblock P(CL-Br)_10_-*b*-PEG-*b*-P(CL-Br)_10_. **(D)** ^1^H NMR spectrum of pentablock PCL_10_-*b*-P(CL-Br)_10_-*b*-PEG-*b*-P(CL-Br)_10_-*b*-PCL_10_. **(E)** ^1^H NMR spectrum of PCL_10_-*b*-P(CL-NE)_10_-*b*-PEG-*b*-P(CL-NE)_10_-*b*-PCL_10_.

**Figure S3**. **Synthesis and characterization of the cationic pentablock copolymer and its self-assembled nanoparticles. (A)** FTIR spectra of PEG, P(CL-Br)_10_-*b*-PEG-*b*-P(CL-Br)_10_ and PCL_10_-*b*-P(CL-Br)_10_-*b*-PEG-*b*-P(CL-Br)_10_-*b*-PCL_10_. **(B)** GPC traces of PEG, P(CL-Br)_10_-*b*-PEG-*b*-P(CL-Br)_10_, PCL_10_-*b*-P(CL-Br)_10_-*b*-PEG-*b*-P(CL-Br)_10_-*b*-PCL_10_ and PCL_10_-*b*-P(CL-NE)_10_-*b*-PEG-*b*-P(CL-NE)_10_-*b*-PCL_10_. **(C)** Excitation and emission spectra of IR808-labeled cNPs. **(D)** GPC traces of cNPs before and after fluorescence modification.

**Figure S4.** **cNP injection protected mice against LPS-induced ALI. (A, B)** Representative H&E-stained sections and pathological damage scores of mouse lung tissues. Scale bars: 100 μm, n = 6/group. **(C, D)** Representative TUNEL-staining images of mouse lung tissues and semi-quantitative analysis of TUNEL-positive areas. Scale bars: 100 μm, n = 6/group. The data shown are mean ± SE (****p* < 0.001).

**Figure S5.** **cNPs have a favorable safety profile *in vivo*. (A, B)** Representative H&E-staining images of sections and TUNEL staining of heart, liver, spleen, lung, and kidney tissues from mice injected with cNPs alone after 1, 3, 5, or 7 days. Scale bar: 100 μm, n = 6/group. **(C, D, E, F)** The serum ALT, AST, Cr, and Urea levels after injection with cNPs alone at 1, 3, 5, or 7 days. The data shown are mean ± SE (***p* < 0.01, ****p* < 0.001).

**Figure S6**. **cNPs inhibited NET formation and cGAS-STING pathway expression in the lung. (A, B)** Statistics of positive areas for cit-H3 (green), MPO (red), and nuclei (blue). **(C, D, E)** Statistics of the grayscale ratio of cGAS, STING, and p-TBK1 proteins to the internal reference in lung tissues (fold change to Sham group). n = 3/group.  **(F, G)** IHC-staining images of lung tissues for F4/80 expression and statistics of positive areas (yellowish-brown color). Scale bars: 100 μm, n = 6/group. The data shown are mean ± SE (**p*< 0.5, ***p*< 0.01, ****p* < 0.001).

**Figure S7. cNPs suppressed the activation of the STING pathway mediated by internalized cfDNA in RAW264.7 macrophages. (A, B, C)** Statistics of the grayscale ratio of STING, p-TBK1, and p-IRF3 proteins to the internal reference in macrophages (fold change to Sham group). n = 4/group. **(D)** The protein expression levels of p-TBK1, TBK1, p-IRF3, and IRF3 of four groups in RAW264.7 macrophages as determined by western blotting after stimulating with cfDNA or poly(dA:dT) , with or without cNPs. **(E, F)** Statistics of the grayscale ratio of p-TBK1/TBK1 and p-IRF3/IRF3 in macrophages (fold change to Sham group), n = 4/group. The data shown are mean ± SE (**p*< 0.5, ***p* < 0.01, ****p* < 0.001).
